# A PMMA-Based Microfluidic Device for Human Sperm Evaluation and Screening on Swimming Capability and Swimming Persistence

**DOI:** 10.3390/mi11090793

**Published:** 2020-08-21

**Authors:** Yimo Yan, Haoran Liu, Boxuan Zhang, Ran Liu

**Affiliations:** 1 Department of Biomedical Engineering, School of Medicine, Tsinghua University, Beijing 100084, China; yym18@mails.tsinghua.edu.cn (Y.Y.); lhrmachinery@163.com (H.L.); zbx19@mails.tsinghua.edu.cn (B.Z.); 2 Tsinghua Shenzhen International Graduate School, Tsinghua University, Shenzhen 518055, China

**Keywords:** sperm, assisted reproductive technology, motility

## Abstract

The selection of high-quality sperm is essential to the success of in vitro fertilization (IVF). As human cervical mucus has a high viscosity, without enough swimming persistence, human sperm clouds cannot arrive at the ampulla to fertilize the egg. In this study, we used swimming capability and motion characteristics that are known to be associated with fertilization ability to evaluate the quality of sperm. Here, a clinically applicable polymethyl methacrylate (PMMA)-based microdevice was designed and fabricated for sperm evaluation and screening for swimming capability and persistence in a viscous environment. In this study, we applied methylcellulose (MC) to mimic the natural properties of mucus in vivo to achieve the selection of motile sperm. Sperm motion was recorded by an inverted microscope. The statistical features were extracted and analyzed. Hundreds of sperm in two treated groups with different concentrations of MC and one control group with human tubal fluid (HTF) media were video recorded. This device can achieve a one-step procedure of high-quality sperm selection and achieve the quantitative evaluation of sperm swimming capability and persistence. Sperm with good swimming capability and persistence may be more suitable for fertilization in a viscous environment. This microdevice and methods could be used to guide the evaluation of sperm motility and screening in the future.

## 1. Introduction

Infertility affects more than 70 million couples worldwide, 30% of infertility cases are caused solely by male factor infertility, and 50% of infertility cases include a combination of male and female factor infertility [1,2]. One of the most prominent causes of male infertility is a decrease in sperm quality [3]. Medical institutions usually evaluate sperm health based on count, vitality, motility, DNA integrity, and morphology, among other characteristics. For humans, successful fertilization also relies on the timely arrival of spermatozoa and egg, as sperm viability can be prolonged within vivo environment and its inactivation can be delayed. The ability to obtain a subpopulation of sperm that is suitable for fertilization is essential in sperm cryopreservation [4,5,6,7], in vitro fertilization (IVF) [8,9,10,11,12,13], intracytoplasmic sperm injection [14], and artificial insemination [15].

The methods used to select sperm for assisted reproductive technology (ART) need several sperm washing steps to acquire healthy sperm, and most protocols of separating sperm are based on their density or motility differences to acquire the motile sperm. However, the probability of DNA damage is increased via these ways. In recent decades, microfluidic systems have been utilized in the field of ART to assist with sperm sorting. Recent studies have focused on developing a simple and cost-effective device for motile sperm selection. Huang [16], Fu [17], Cho [18], Schuster [19], and Matsuura [20] designed microfluidic chips that can successfully obtain motile sperms. Collectively, these previous studies made use of the principle that motile sperm can swim through laminar interfaces to achieve a selection of high-motility sperm in vivo.

As for humans, approximately 300 million sperm are ejaculated into the vagina during sexual intercourse, however, only about ~one of every million sperm succeed into the fallopian tube [21,22,23]. Additionally, sperm must undergo a process of capacitation, as only the capacitated sperm can finally reach the oocyte membrane and achieve fertilization. These facts, together with the tiny dimensions of the sperm in comparison to the long journey in the fallopian tube, make the arrival of the sperm to the fertilization site improbable. Therefore, it is believed that sperm must be guided during that process. So far, three such guidance mechanisms have been proposed, such as chemotaxis [24,25,26,27], where sperm swim up a chemoattractant (biochemical) gradient. Xie et al. [28] obtained motile sperm using a microfluidic chip with a modified straight pipe length and width. Additionally, they selectively cultured cumulus cells in a bi-branch channel to generate a chemoattractant-forming chemical gradient. Sperm chemotaxis was represented by the proportions of sperm swimming towards the different branches. Approximately 10% of sperm were found to be chemotactically responsive in their experiment. As for thermotaxis [29,30,31,32], sperm can swim up a temperature gradient, and it also has been demonstrated to be an important criterion in sperm quality evaluation. Li et al. [33] employed a microfluidic system with an interfacial valve. A thermal gradient was established between two branches and precisely controlled by an external temperature gradient control system. Thermotaxis sperm gathered into a higher-temperature branch, and non-thermotaxis sperm swam non-preferentially toward both branches. With the device, about 6–11% of motile sperm had a response to the thermal gradient. Both chemotaxis and thermotaxis are short-range mechanisms; however, rheotaxis [34,35,36] is a long-range mechanism in which sperm can swim up against a flow. Zaferani et al. [37] designed a microfluidic device that can non-invasively and passively separate motile sperm from the semen sample. This device relied on the rheotactic behavior of sperm and was able to corral motile sperm.

To a certain extent, these studies were able to obtain a subpopulation of sperm that performs relatively well in certain aspects. However, these studies did not consider the selection effects of the female micro-environment, which will vary during real fertilization scenarios. In the natural fertilization process, sperm are always surrounded by the viscous media; the viscous media directly affects sperm motility, thereby affecting the fertilization process. Hyun et al. [38] further studied the influences of liquid viscosity on spermatozoa motility, and they demonstrated that viscosity only affects the mechanical properties rather than energetics under viscous conditions. Previous studies have already evaluated methylcellulose (MC) as a preferred medium for mimicking the natural properties of mucus in sperm migration tests [39]. Hence, we further explored the screening effect of different values of viscosity on sperm and performed a quantitative analysis of sperm motility under multiple viscosity environments. Compared with current clinical selection methods such as swim-up and density gradient centrifugation [40,41], this polymethyl methacrylate (PMMA)-based microfluidic device is easier to operate and reusable; it can be used clinically to select the ideal sperm, which has the potential to improve reproductive success rate outcomes.

In the real fertilization process, sperm not only require enough forward swimming capability in the viscous fluid but also need enough swimming persistence to ensure they finally arrive at the fertilization site. However, thus far, none of the scholars have made a comprehensive evaluation of sperm based on swimming capability, swimming persistence, and sperm inactivation rate in a viscous medium. Moreover, the viscosity of the fluid in the female genital tract changes with time and is influenced by many factors such as the secretion of ovarian steroid hormones. The migration of sperm will be influenced by the viscosity of the fluid. Different viscosity conditions of fluid will influence the movement and swimming pattern of sperm, which will have a further impact on sperm metabolism.

However, recently, viscosity has not received enough consideration as a major reference on human reproduction. This paper studies the motility parameters of sperm in a multi-viscosity environment through a circular symmetric chip and aims to find a more prepotent sperm subpopulation that has good swimming capability and swimming persistence. A quantitative analysis of sperm motility parameters was performed to study their responding mechanism under different viscosity conditions. Finally, we hope our study can be used to improve the sperm motility assessment and guide the evaluation of sperm motility and screening.

## 2. Materials and Experimental

### 2.1. Chip Fabrication

**Figure 1:**
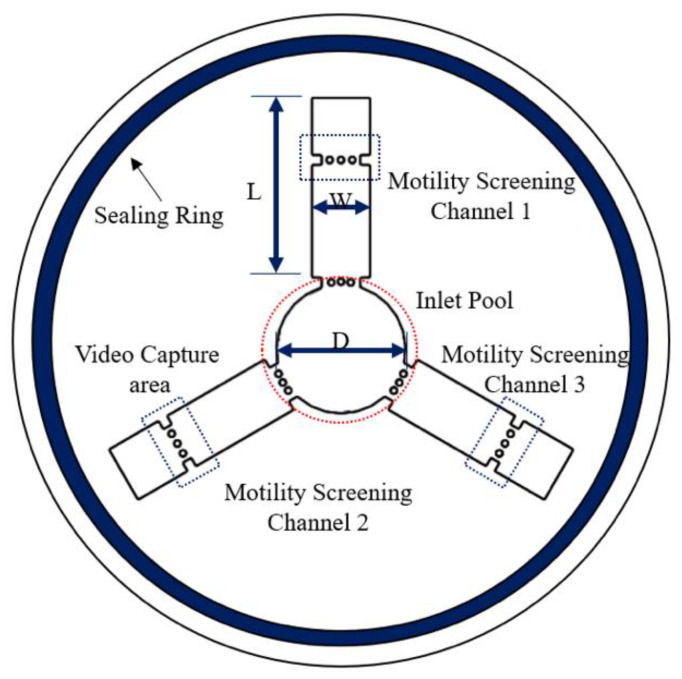
Schematic diagram of the device. Microfluidic device for human sperm evaluation and screening of swimming capability and swimming persistence; W and L represent the width and length of the channel, respectively. D represents the diameter of the inlet pool (the dimensions of the device are shown in Figure S1).

Figure 1 shows the structural schematic diagram of the circular symmetric chip. The inlet pool is used for adding semen samples, and motility screening channels 1, 2, and 3 are used for sperm screening. There are two trapezoid structures combined with three micro-columns at the junction of each straight line and the circular chamber. These structures are used to prevent the mixing of the adjacent media. The circular structure in the middle of each straight pipe is a marker for video capture areas. A cover slide can be attached to it, preventing chip liquid volatilization and environmental interference. The seal ring is used to seal the chip to make the surface wet. The width and length of the channel are 1.8 mm and 6.5 mm, respectively. The diameter of the inlet pool is 4 mm, and the channel depth is 150 microns.

The device was fabricated with polymethyl methacrylate (PMMA). As PMMA material has good optical properties, it is easy to observe under a microscope. It also has outstanding mechanical processing characteristics, which can be milled and laser processed. PMMA has excellent scratch resistance and is able to be processed to a very high gloss finish. Additionally, it also has good biological compatibility, does no harm, and does not disrupt natural biological processes during the experimental procedure.

The microfluidic chip was designed in SolidWorks and the stereolithograph (STL) files were imported into the SRP Player of the engraving machine (Roland, Los Angeles, CA, USA, MX-40) for tool selection, processing parameter design, and cutting. Next, the engraving machine was started, and the VPanel software (Roland, Los Angeles, CA, USA) was used to perform the three-axis positioning of the engraving machine. After completing all the preparations, cutting was performed.

This PMMA chip is molded in one-step and is inexpensive, making it suitable for point-of-care testing (POCT).

### 2.2. Semen Sample Preparation

Human semen samples were collected, processed, and tested in accordance with the WHO guidelines and approved by the Institution Review Board of Tsinghua University, and informed consent was obtained from all subjects. Semen samples were provided by Peking University Third Hospital’s Reproductive Medicine Center. Semen was obtained from healthy donors by masturbation after approximately three days of sexual abstinence and incubated for 30 min at 37 °C to allow liquefaction. The information for this study remained confidential and within the institution. Prior to the experiment, the semen samples were purified via two-layer density centrifugation (300× *g* for 20 min) using PureSperm^®^40 and PureSperm^®^80 (Nidacon, Mölndal, Sweden). The supernatant was discarded, and the sperm pellet was resuspended and centrifuged again [42] (500× *g* for 10 min) in 4.5 mL of PureSperm^®^ wash (Nidacon, Mölndal, Sweden).

### 2.3. Buffer Preparation

Several solutions with different viscosities were created by adding 5% and 3.4% methylcellulose to the human tubal fluid (HTF). The configuration method of the methylcellulose solution is as follows: a certain quality of methylcellulose powder was gradually added into HTF, which had been heated to a high temperature and continuously stirred. After a while, all the powder entered the solution system, and it was stirred for another 10 min, then the entire solution system became a gel state and lost its fluidity. Next, the gel state system was placed in an ice bath and continuously stirred, the system gradually became liquid, but a small amount of MC did not enter into the solution system. The heating and ice bath processes were repeated until all the methylcellulose powder was dissolved.

HTF was used to dispose of the methylcellulose viscous liquid. Based on our experiments, it was found that the sperm in Phosphate-buffered saline (PBS) and pure water cannot maintain its motility and vitality well, but it can be done in HTF. According to previous experiments, the sperm in the chip filled with hyaluronic acid (HA) solution lost vitality easily, and the number of sperm that swam into the end of the channel was scarce. However, the problem was solved when the media changed to the MC solution.

### 2.4. Viscosity Change Assessment

To observe the swimming pattern of sperm in different viscosity conditions, the medium viscosity in the video capture area for each straight pipe needs to remain constant over time, which was critical to the success of the experiment.

Therefore, the media need to remain stable in the chip during the experiment. In this study, a multi-viscosity (3) solution was used simultaneously on a single chip. Due to molecular motion at the junction of the straight pipeline and the central circular chamber, there would be a mixing of liquids, leading to changes in the viscosity of the straight pipe. To prevent this phenomenon, numerical simulation of chemical diffusion was performed to predict the viscosity change and further optimize the chip structure design. It was also required to determine the range of changes in viscosity attained in the straight pipeline for the entire experiment.

**Figure 2:**
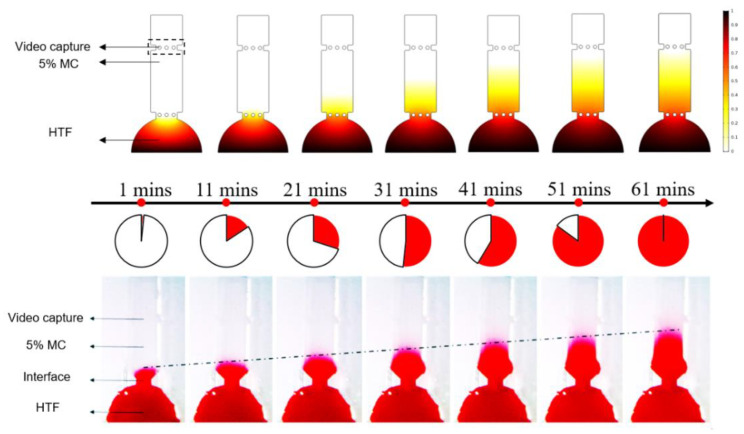
Solution-diffusion experiment and simulation. A black dashed line indicates the front end of the red liquid.

Computation fluid dynamics and chemical diffusion simulations were performed using COMSOL 5.3a (COMSOL AB, Burlington, MA, USA). A two-dimensional finite element geometry was built based on variable parameters and analyzed using a laminar flow field and chemical diffusion field. As three channels, 1, 2, and 3, were united, in order to prevent uncontrolled convective mixing, we added the structure of micro-columns at the junction of each straight line and the circular chamber. Consequently, the transfer of various viscosity media among channels occurred only by diffusion in the restricted area and media with low diffusion coefficients stayed in their respective channels. We performed the experiments of solution diffusion and numerical simulation simultaneously to verify our assumptions. Color diffusion was used to approximately simulate liquid mixing at the junction. A 5% MC solution was first added into the straight pipe, then stained HTF solution was added into the central circular chamber. The chip was further sealed using a sealing ring and a cover slide. Then, a microscope (XTZ-CT, Shanghai Optical Instrument Factory Six, Shanghai, China) was used to record the diffusion of red liquid (stained HTF solution) in the straight pipe for 60 min. The experiment and simulation results of the diffusion of stained HTF solution over time in the straight channel is shown in Figure 2. As shown in Figure 2, the simulation results were basically consistent with the actual experimental results. The experimental results showed that color diffusion was approximately uniform in the straight pipe for 60 min and did not reach the video capture area. Therefore, we consider the circular symmetric chip to be stable for 60 min.

### 2.5. Experimental Process

**Figure 3:**
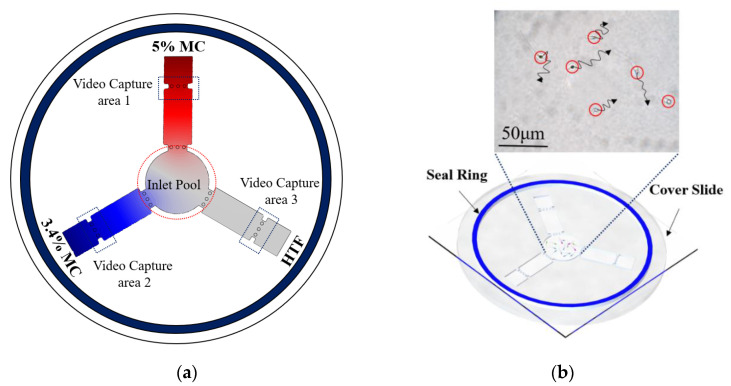
Experimental procedure for the sperm motility test. (**a**) 5%, 4% methylcellulose (MC) solution, and human tubal fluid (HTF) solution, respectively, was added to different straight pipes. Simulation results show the final diffusion condition. (The color intensity shows the different concentration of MC solution) (**b**) 1 mL semen sample was pipetted into the inlet of the microfluidic device (red circles show the position of motile sperm), then a cover slide was placed on the microfluidic chip, and a performance motility analysis of sperm was performed.

Each of the three straight pipes was filled with either 5% or 3.4% MC solution or HTF solution, and the semen sample was added to the central circular chamber. Figure 3a shows the final diffusion condition after three different media were pipetted into different channels. The various colors show MC solutions at different concentrations. Figure 3b shows the chip after adding the semen samples, and images were taken at each of the screening areas every 4 min to track sperm movement. After adding the semen sample, we put a cover slide on the PMMA device, then the sealing ring was quickly wetted and the chip was sealed with the cover. The chip system was placed in the inverted microscope (DMIRB, Leica, Wetzlar, Germany), and videos were recorded at each of the pipes. We independently replicated each experiment two times, with sperm samples from four donors.

## 3. Results and Discussion

### 3.1. Sperm Swimming Capability Distribution

**Figure 4:**
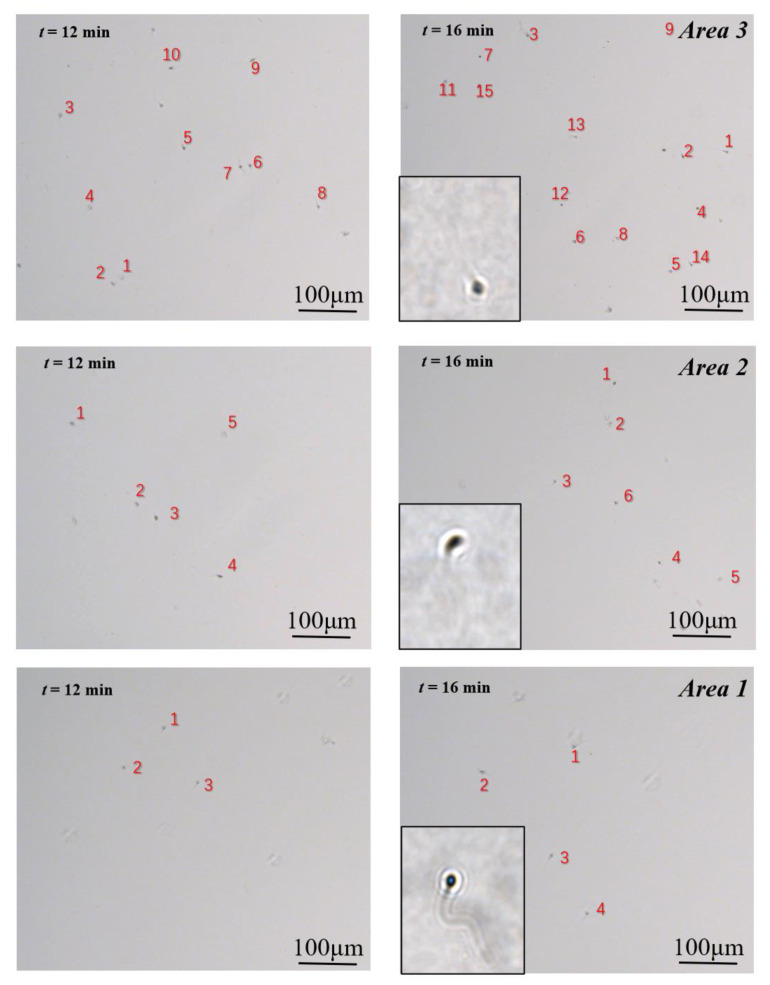
Motile sperm images in the three video capture regions at 12 min and 16 min. The number of different grades of sperm (A, B, and C) can be obtained through calculation. Number of Sperm A = Number of sperm in Area 1; Number of Sperm B = Number of sperm in Area 2—Number of sperm in Area 1; Number of Sperm C = Number of sperm in Area 3—Number of sperm in Area 2.

In this paper, we introduced a new assessment criterion: swimming capability. Compared with sperm motility, which refers to the movement and swimming of sperm, it puts more emphasis on the swimming ability of the sperm, in order to penetrate the viscous fluid. The images of sperm acquired at different time frames (12 min and 16 min) were presented in Figure 4. During the same period, there were fewer motile sperm in the zone filled with a 5% MC solution (named Area 1) than in the zone filled with a 3.4% MC solution (named Area 2). In addition, the greatest number of motile sperm was in the zone filled with HTF solution (named Area 3).

**Figure 5:**
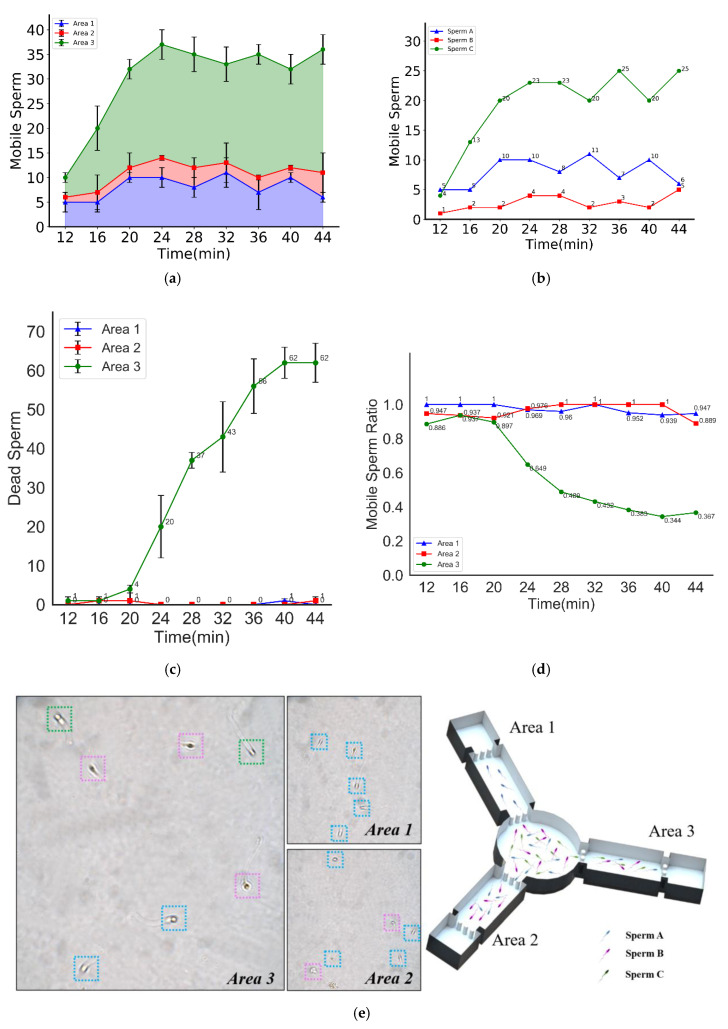
Sperm selected by swimming capability and swimming persistence. (**a**) The numbers of motile sperm in different areas vs. time. (**b**) The number of sperm of different grades vs. time. (**c**) The number of dead sperm in different areas vs. time. (**d**) The ratios of motile sperm (calculated by the ratio of the number of motile sperm to the total number of sperm) in different areas vs. time. (**e**) Schematic diagram of sperm classification, the different colored boxes represent the different grades of sperm (A, B, and C).

Figure 5a shows the number of motile sperm in each video capture area changed with time. As seen from the chart, Area 1 had the fewest number of motile sperm, followed by Area 2, and Area 3 had the greatest number of motile sperm in the images.

It was found that a lower media viscosity resulted in less resistance for motile sperm, which means more sperm could penetrate the media and swim to the corresponding video capture zone. In contrast, a higher viscosity resulted in greater resistance for motile sperm. If the sperm do not have the capability to resist the viscous medium, they cannot successfully penetrate the medium to reach the video capture zone. Therefore, the number of motile sperm in the 5% MC solution (as shown in Area 1) is the lowest, while the greatest number of sperm are found in the HTF solution (as shown in Area 3).

In addition, as seen from the chart, the number of motile sperm in each video capture area was approximately constant after 20 min. Thus, the proportion of motile sperm in Areas 1, 2, and 3 was also kept approximately constant.

Hence, in this study, according to their respective swimming capability, the motile sperm were divided into three groups, namely, Sperm A, Sperm B, and Sperm C: sperm that can overcome the resistance of the viscous medium in Area 1 are named Sperm A, and sperm that can overcome the resistance of the viscous medium in Area 2 but cannot swim in Area 1 are named Sperm B. Finally, sperm that can overcome the resistance in Area 3 but cannot swim in Area 2 are named Sperm C.

Due to the symmetry of the chip structure, each of the motile sperm from the circular chamber swam with equal probability to each of the straight pipes. Based on our prior experiments, unlike thermotaxis and chemotaxis, sperm have nearly no preferences for specific viscosity solutions. Therefore, the probability will remain the same for sperm traveling into different pipes. The experiments needed to be performed for a sufficiently long period so that the number of Sperm A in all three video capture areas would be the same. Similarly, the same number of Sperm B were in Area 2 and Area 3, and Sperm C could only swim in Area 3 due to their poor swimming capability. In summary, Area 1 contained only Sperm A, Area 2 contained not only a similar number of Sperm A as in Area 1 but also Sperm B, and Area 3 contained not only the same number of Sperm A and Sperm B as in Area 2 but also Sperm C.

The motile sperm swimming capability distribution in the semen sample could be obtained through calculation. Figure 5b shows the swimming capability distribution of motile sperm. As seen from the chart, the number of Sperm A, Sperm B, and Sperm C in the semen sample remained approximately constant after 20 min. Additionally, the proportion of Sperm A, Sperm B, and Sperm C, namely, the swimming capability distribution of the motile sperm, was also kept approximately constant.

### 3.2. Average Straight-Line Velocity of Motile Sperm

**Figure 6:**
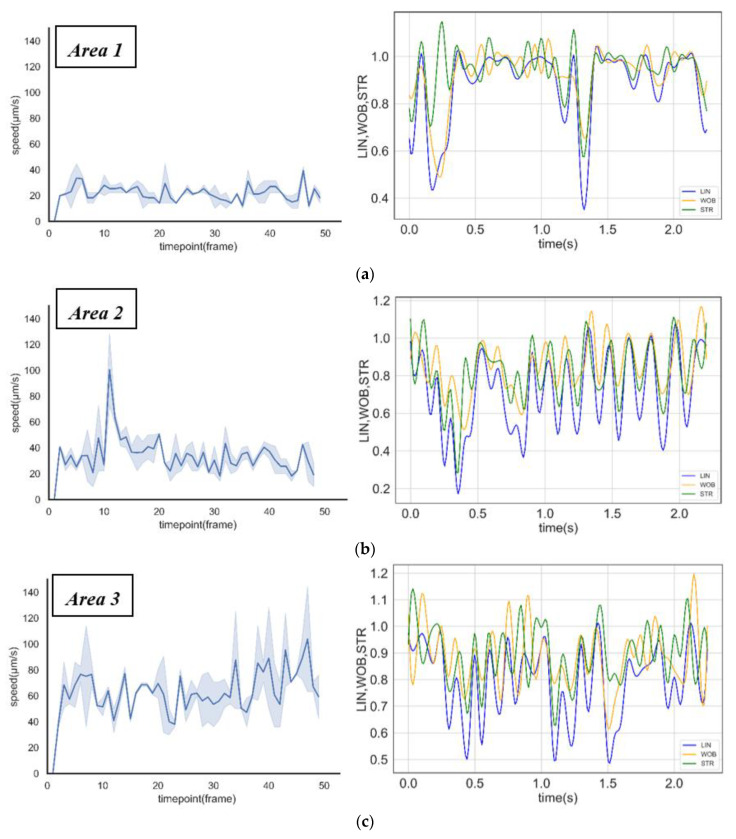
Sperm motility analysis in different areas. (**a**–**c**) Straight-line velocity distribution and motion behaviors (linearity, wobble, and straightness) of sperm in Area 1, Area 2, and Area 3.

A research study by Eamer et al. [43] suggests that human sperm swim slower and straighter as the viscosity of the swimming medium increases. In this study, the motility differences were caused by the change in viscosity. Based on the tracking of the specific grade of sperm, it was found that Sperm A could swim rapidly from the central circular chamber to Area 1. The count soon reached a dynamic equilibrium state; Sperm B swimming towards Area 2 took a longer time to attain a dynamic equilibrium state, and Sperm C took the longest time during the same process. The velocity distribution and motility parameters of sperm in different areas were shown in Figure 6a–c. When sperm swam into the high viscosity area (such as Area 1 and Area 2), their velocity decreased, and their motion behavior changed intensively (the motion parameters vs. time were shown in Figure 6a–c). This indicated that the sperm head began to wobble rapidly. These changes in motility and motion behaviors were the response mechanism of sperm in different viscosities.

### 3.3. Sperm Screening for IVF

Due to energy consumption, motile sperm may die or lose their motility during the swimming process. This phenomenon was noted in our study. Figure 5c shows the change in the number of dead sperm with time in each video capture area. It was observed in Areas 1 and 2 that there were basically no dead sperm throughout the whole experimental process. In Area 3, however, there were a few dead sperm within the first 20 min, but a more notable number of dead sperm appeared after 20 min. The number of dead sperm increased proportionally with time from 20 min to 40 min, followed by only a slight increase after 40 min.

Figure 5d shows changes in the proportion of motile sperm to the total number of sperm in each video capture area with time. It was found that the ratio of motile sperm was approximately 100% in Area 1 and approximately 90% in Area 2 and Area 3 before 20 min, but in Area 3 the ratio of motile sperm declined rapidly to 36% from 20 min to 36 min. The average percentage rate of decrease in motile sperm, namely, the average sperm inactivation rate, in Area 3 was 3.38% per min.

It was found that some motile sperm in the semen sample lost their swimming capacity easily, and these sperm were probably not equally distributed in Sperm A, Sperm B, and Sperm C. According to experimental results, the stronger the swimming capability, the longer its motility. Accordingly, Sperm A and Sperm B both had good swimming capability and more durability, while Sperm C had poor swimming capability, cannot swim with the same durability and can easily lose their motility. Sperm C might not be able to arrive at the fertilization site in the female genital tract successfully. The inference drawn from these experimental results was that a subpopulation of sperm existed in the semen sample that not only has excellent swimming capability and swimming persistence but also can swim through viscous media rapidly such as Sperm A in our study. Hence, these sperm might be more suitable for achieving fertilization in the female genital tract.

## 4. Conclusions

This paper describes a multi-viscosity circular symmetric chip designed for sperm evaluation and screening for swimming capability, swimming persistence, and inactivation rate. It has successfully been shown that a specific type of sperm may be more suitable for fertilization in the female genital tract.

As per the experimental results, it was found that sperm with poor swimming capability lost their motility more easily in semen, and this kind of sperm also had poor swimming persistence. It is difficult for such sperm to arrive at the fertilization site successfully. We also found another type of sperm that had good swimming capability and swimming persistence, these sperm could swim quickly and be more durable, which may better enable them to reach the fertilization site. In other words, these sperm were more suitable for fertilization.

In male reproductive health diagnosis, semen quality evaluation indices include VSL distribution, sperm morphology, semen pH, sperm density, sperm volume, etc. However, the evaluation of swimming capacity and swimming persistence which were proposed in our study can further improve sperm motility assessment. These parameters could further reflect the fertilization ability of sperm to a certain degree, and it may indicate specific reference values in the diagnosis of male reproductive health.

Furthermore, in view of the sperm found in our experiment that had a superior potential for fertilization, the experimental method used in our study could be combined with traditional screening methods in assisted reproductive technology to obtain sperm with good quality for IVF and future scientific research.

## Supplementary Materials

The following are available online at https://www.mdpi.com/2072-666X/11/9/793/s1, A PMMA-Based Microfluidic Device for Human Sperm Evaluation and Screening on Swimming Capability and Swimming Persistence. Figure S1. Dimensions of the PMMA-based microfluidic device. (in mm).
