# Supplementary material for: A PMMA-Based Microfluidic Device for Human Sperm Evaluation and Screening on Swimming Capability and Swimming Persistence
Source: Micromachines (Basel). 2020 Aug 21;11(9):793. doi: 10.3390/mi11090793 (PMC7570091; doi:10.3390/mi11090793)
Supplement: Supplementary file 1 [file micromachines-11-00793-s001.zip › supplementary proofreading/micromachines-863111 - Authorship Change Form.docx]

Micromachines **— Change of Authorship Form**

(Must be Completed and Signed by ALL Authors)

Manuscript ID: micromachines-863111

Manuscript Title: A PMMA-Based Microfluidic Device for Human Sperm Evaluation and Screening on Swimming Capability and Swimming Persistence

Description of the change: (new author have been added)

**Original Authorship**

LIST ALL AUTHORS in the same order as the original (first) submission. For more than 10 use an extra sheet.

|  | Name | Affiliation |
| --- | --- | --- |
| author (1) | Yimo Yan | Department of Biomedical Engineering, School of Medicine, Tsinghua University, Beijing 100084, China |
| author (2) | Haoran Liu | Department of Biomedical Engineering, School of Medicine, Tsinghua University, Beijing 100084, China |
| author (3) | Ran Liu | Tsinghua Shenzhen International Graduate School, Tsinghua University, Shenzhen 518055, China |
| author (4) |  |  |
| author (6) |  |  |
| author (7) |  |  |
| author (8) |  |  |
| author (9) |  |  |
| author (10) |  |  |

**New Authorship**

All authors must sign below agreeing to the new changes in authorship. The authorship order must match the new title page of the manuscript. Signatures below certify compliance with the author responsibilities on the next page. List ALL AUTHORS in the same order as the new version.

|  | Title(Mr./Ms./Mrs./Dr./Prof.) | Name | Email | Affiliation | Signature&Date |
| --- | --- | --- | --- | --- | --- |
| author (1) |  |  |  |  |  |
| author (2) |  |  |  |  |  |
| author (3) |  |  |  |  |  |
| author (4) |  |  |  |  |  |
| author (5) |  |  |  |  |  |
| author (6) |  |  |  |  |  |
| author (7) |  |  |  |  |  |
| author (8) |  |  |  |  |  |
| author (9) |  |  |  |  |  |
| author (10) |  |  |  |  |  |

Please list all the author’s Contribution here:

| Author’s Contribution |
| --- |
| author (1) |
| author (2) |
| author (3) |
| author (4) |
| author (5) |
| author (6) |
| author (7) |
| author (8) |
| author (9) |
| author (10) |

Contributor Roles Taxonomy (CRediT)

| Conceptualization | Ideas; formulation or evolution of overarching research goals and aims. |
| --- | --- |
| Data curation | Management activities to annotate (produce metadata), scrub data and maintain research data (including software code, where it is necessary for interpreting the data itself) for initial use and later re-use. |
| Formal analysis | Application of statistical, mathematical, computational, or other formal techniques to analyze or synthesize study data. |
| Funding acquisition | Acquisition of the financial support for the project leading to this publication. |
| Investigation | Conducting a research and investigation process, specifically performing the experiments, or data/evidence collection. |
| Methodology | Development or design of methodology; creation of models. |
| Project administration | Management and coordination responsibility for the research activity planning and execution. |
| Resources | Provision of study materials, reagents, materials, patients, laboratory samples, animals, instrumentation, computing resources, or other analysis tools. |
| Software | Programming, software development; designing computer programs; implementation of the computer code and supporting algorithms; testing of existing code components. |
| Supervision | Oversight and leadership responsibility for the research activity planning and execution, including mentorship external to the core team. |
| Validation | Verification, whether as a part of the activity or separate, of the overall replication/reproducibility of results/experiments and other research outputs. |
| Visualization | Preparation, creation and/or presentation of the published work, specifically visualization/data presentation. |
| Writing - original draft | Preparation, creation and/or presentation of the published work, specifically writing the initial draft (including substantive translation). |
| Writing - review & editing | Preparation, creation and/or presentation of the published work by those from the original research group, specifically critical review, commentary or revision – including pre- or post-publication stages. |
