# Supplementary material for: A PMMA-Based Microfluidic Device for Human Sperm Evaluation and Screening on Swimming Capability and Swimming Persistence
Source: Micromachines (Basel). 2020 Aug 21;11(9):793. doi: 10.3390/mi11090793 (PMC7570091; doi:10.3390/mi11090793)
Supplement: Supplementary file 1 [file micromachines-11-00793-s001.zip › supplementary proofreading/micromachines-863111 supplementary.docx]

Supplementary materials: A PMMA-Based Microfluidic Device for Human Sperm Evaluation and Screening on Swimming Capability and Swimming Persistence


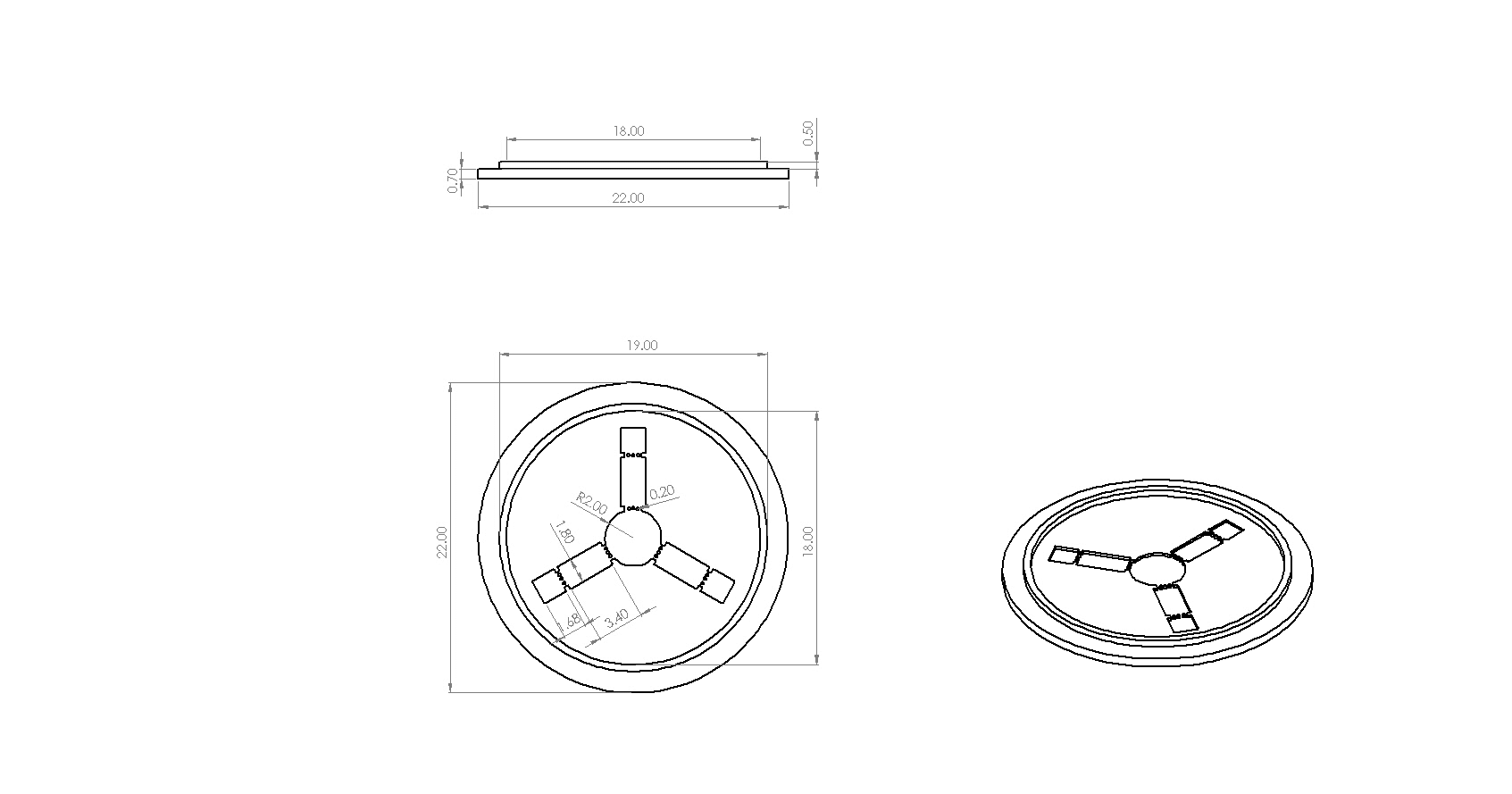


**Figure S1.** Dimensions of the PMMA-based microfluidic device. (in mm).
